# Effects of Fe oxide on N transformations in subtropical acid soils

**DOI:** 10.1038/srep08615

**Published:** 2015-02-27

**Authors:** Xianjun Jiang, Xiaoping Xin, Shiwei Li, Junchao Zhou, Tongbin Zhu, Christopher Müller, Zucong Cai, Alan L. Wright

**Affiliations:** 1 College of Resources and Environment, Southwest University, 2 Tiansheng Road, Beibei, Chongqing 400715, China; 2 School of Geography Sciences, Nanjing Normal University, Nanjing 210047, China; 3 Key Laboratory of Reservoir Aquatic Environment, Chongqing Institute of Green and Intelligent Technology, Chinese Academy of Science, Chongqing 400714, China; 4 Department of Plant Ecology (IFZ), Justus-Liebig University Giessen, Heinrich-Buff-Ring 26, 35392 Giessen, Germany; 5 School of Biology and Environmental Science, University College Dublin, Ireland; 6 Everglades Research & Education Center, University of Florida, Belle Glade, FL 33430, United States

## Abstract

Subtropical ecosystems are often characterized by high N cycling rates, but net nitrification rates are often low in subtropical acid soils. NO_3_^−^-N immobilization into organic N may be a contributing factor to understand the observed low net nitrification rates in these acid soils. The effects of Fe oxide and organic matter on soil N transformations were evaluated using a ^15^N tracing study. Soil net nitrification was low for highly acidic yellow soil (Ferralsols), but gross ammonia oxidation was 7 times higher than net nitrification. In weakly acidic purple soil (Cambisols), net nitrification was 8 times higher than in Ferralsols. The addition of 5% Fe oxide to Cambisols, reduced the net nitrification rate to a negative rate, while NO_3_^−^-N immobilization rate increased 8 fold. NO_3_^−^-N immobilization was also observed in Ferralsols which contained high Fe oxides levels. A possible mechanism for these reactions could be stimulation of NO_3_^−^-N immobilization by Fe oxide which promoted the abiotic formation of nitrogenous polymers, suggesting that the absence of net nitrification in some highly acid soils may be due to high rates of NO_3_^−^-N immobilization caused by high Fe oxide content rather than a low pH.

Soils in subtropical and tropical forests are generally characterized by rapid N cycling rates and high N availability^1^,^2^,^3^. However, a few studies indicated that nitrification seems to be absent or a minor process in subtropical acid soils^4^,^5^,^6^,^7^. This interpretation was supported by two observations: one is that NO_3_^−^ concentrations are often consistently low in these soils, and that net nitrification rates are low or even negative during incubation assays of soil samples^6^,^7^,^8^,^9^. However, this does not necessarily mean that the nitrification does not occur in these subtropical acid soils. Alternatively, it is possible that NO_3_^−^-N produced in these soils during nitrification (ammonia plus organic N oxidation) is immobilized through both abiotic and biotic processes as for observed in coniferous forest soils^10^. For example, high rates of NO_3_^−^-N immobilization into organic N can be responsible for N enrichment in subtropical acid forest soils^3^ which could protect the soil N from leaching. Furthermore, NO_3_^−^-N can be converted readily to other inorganic forms through denitrification or dissimilatory NO_3_^−^ reduction to NH_4_^+^ (DRNA). Previous studies also suggested that N immobilization can be controlled by the concentration of available C^11^ and available inorganic N^12^,^13^. Nugroho et al.^14^ observed that net nitrification rates in acid soils can vary about three fold at similar soil pH but with different organic matter content. Of course, both biotic and abiotic processes may be significant or one process may dominate, depending on the soil conditions. However, the causes of variation of N immobilization between soil types are not well known^15^.

Subtropical and tropical acid soils are often characterized by high levels of Fe oxides. It has been hypothesized that Fe may play a key role in regulating NO_3_^−^ immobilization in acid forest soils^16^. Likewise, the inhibition of nitrification by high amounts of iron from pyrite (FeS_2_) can also occur^17^. Shindo and Huang^18^ found that Mn oxide promoted the abiotic formation of nitrogenous polymers. Since Fe and Mn oxides have similar chemical properties, it is possible that Fe oxide can also promote organic N formation from NO_3_^−^ Therefore, Fe oxide may be a primary player in N transformations in subtropical acid soils and may help to explain the low NO_3_^−^ concentrations often observed. Here, we hypothesized that nitrification does occur in subtropical acid soils, and NO_3_^−^-N produced during the nitrification process is immobilized quickly by Fe oxide. A weakly acidic purple soil (Cambisols, Chromic) and a highly acidic yellow soil (Ferralsols, Xanthic) were selected to test this hypothesis. Both soils formed from the same parent material in the same climate but had different Fe oxide content. The effects of Fe oxide or organic substances addition on N transformations in these soils were estimated using a ^15^N tracing method and N transformation model^19^.

## Results

### Effects of Fe oxide or organic substances addition on NH_4_^+^-N and NO_3_^−^-N concentrations during incubation

**Figure 1:**
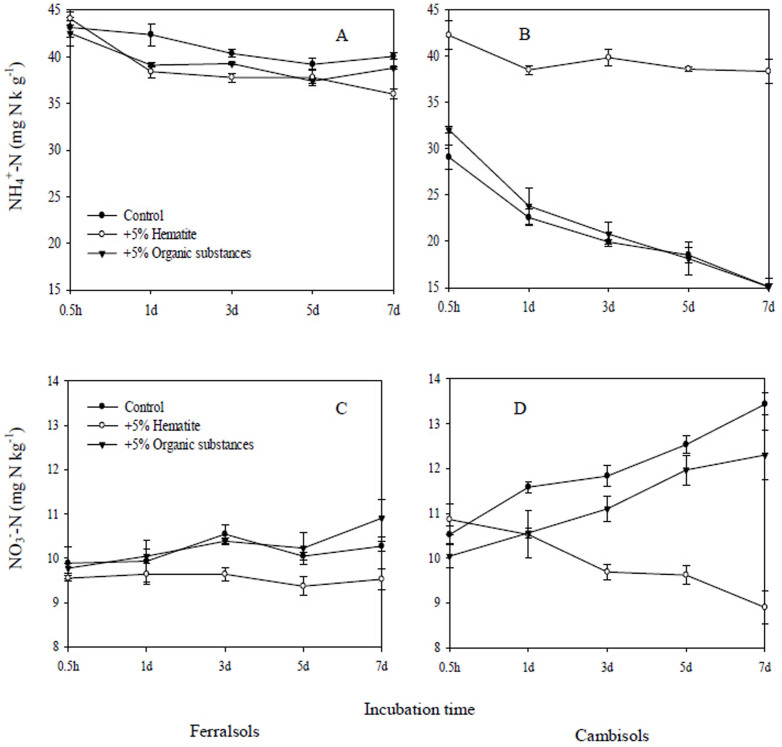
Effects of Fe oxide or organic substances addition on NH_4_^+^-N and NO_3_^−^-N dynamics during 7-day's by ^15^N tracing. (NH_4_^+^-N concentration was measured following the addition of 50 mg kg^−1^ ^15^NH_4_NO_3_; NO_3_^−^-N concentration was measured following the addition of 50 mg kg^−1^ NH_4_^15^NO_3_). Error bars represent standard deviation, n = 3.

Half an hour after 50 mg kg^−1^ ^15^NH_4_NO_3_ spiking, NH_4_^+^-N concentrations for the Ferralsols control averaged 43.1 ± 1.02 mg N kg^−1^. Organic substance or Fe oxide addition did affect NH_4_^+^-N concentrations significantly. During the 7 days incubation period, NH_4_^+^-N concentrations did not change significantly for the Ferralsols control and its two treatments (Figure 1A). However, NH_4_^+^-N dynamics for the Cambisols differed from Ferralsols, as Fe oxide and organic substances addition caused higher significant effects for Cambisols than Ferralsols. At half an hour, after 50 mg kg^−1^ ^15^NH_4_NO_3_ was introduced, NH_4_^+^-N concentrations decreased rapidly for the Cambisols control, and further declined during the 7-day incubation (*p* < 0.05). A similar trend was observed when organic substances were added to Cambisols. However, Fe oxide addition did not change NH_4_^+^-N concentrations throughout the 7-day incubation (Figure 1B).

Half an hour after the addition of 50 mg kg^−1^ NH_4_^15^NO_3_, NO_3_^−^-N concentrations for the Ferralsols control averaged 9.89 ± 0.36 mg NO_3_^−^-N kg^−1^. Addition of organic substances or Fe oxide did not affect NO_3_^−^-N concentrations. During the 7 days incubation period, NO_3_^−^-N concentrations did not change significantly, and did not respond to Fe oxide or organic substances addition in the Ferralsols control (Figure 1C). Conversely, NO_3_^−^-N dynamics in the Cambisols differed were significantly from the one in the Ferralsols. Half an hour, after 50 mg kg^−1^ NH_4_^15^NO_3_ application, NO_3_^−^-N concentrations for the Cambisols control averaged 10.5 ± 0.20 mg N kg^−1^. NO_3_^−^-N concentrations increased significantly for the Cambisols control and organic substances treatment through time, but decreased significantly in the Fe oxide treatment during the 7 days incubation (Figure 1D).

### Effects of Fe oxide or organic substances addition on N transformation rates

Our results clearly showed that soil net nitrification rates, gross autotrophic nitrification (ammonia oxidation) rates, NH_4_^+^ immobilization rates and NO_3_^−^ immobilization rates were significantly affected by Fe oxide addition.

The net nitrification rate for the Ferralsols averaged 0.05 ± 0.01 mg N kg^−1^ soil day^−1^, which was significantly lower than for Cambisols (0.41 ± 0.04 mg N kg^−1^ soil day^−1^), and the organic substances treatment showed a non-significant decreasing trend in nitrification rates (p > 0.05). However, Fe oxide addition decreased the net nitrification rate significantly from 0.41 ± 0.04 to 0.28 ± 0.03 mg N kg^−1^ soil day^−1^ for Cambisols but not for Ferralsols.

**Figure 2:**
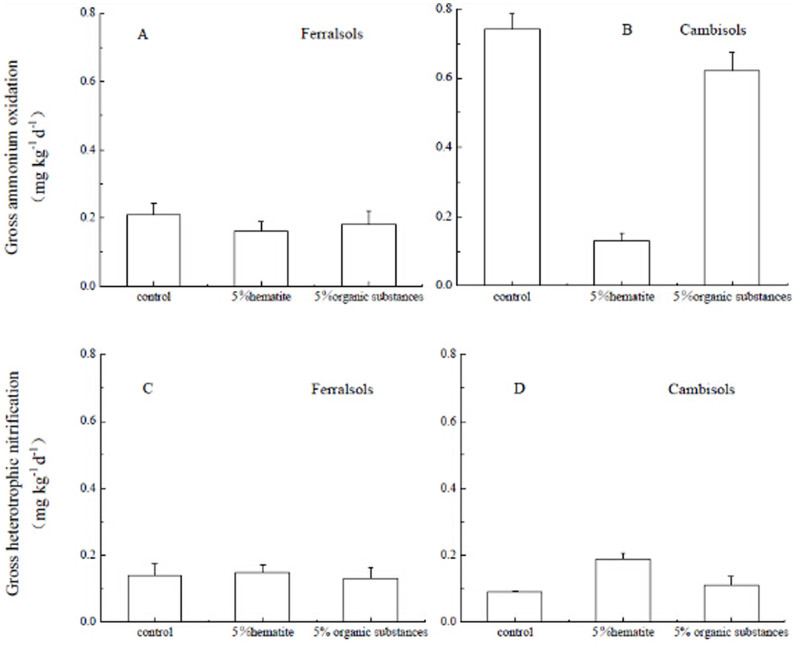
Effects of Fe oxide or organic substances addition on gross heterotrophic nitrification and gross ammonia oxidation in Ferralsols and Cambisols estimated by the ^15^N tracing. Error bars represent standard deviation, n = 3.

The gross ammonia oxidation rates were 0.21 ± 0.03 mg N kg^−1^ day^−1^ and 0.74 ± 0.05 mg N kg^−1^ day^−1^ for the Ferralsols control and Cambisols respectively (p < 0.05) (Figure 2B). Furthermore, Fe oxide addition significantly decreased the gross NH_4_ oxidation rate in Cambisols, whereas the organic substances amendment did not change gross ammonia oxidation (p > 0.05). Neither Fe oxide nor organic substances amendments changed gross NH_3_ oxidation rates in the Ferralsol (Figure 2A).

Fe oxide or organic substances amendments did not change heterotrophic nitrification rates for the Ferralsols (Figure 2C). However, the Fe oxide treatment increased the heterotrophic nitrification rate for Cambisols (Figure 2D).

**Figure 3:**
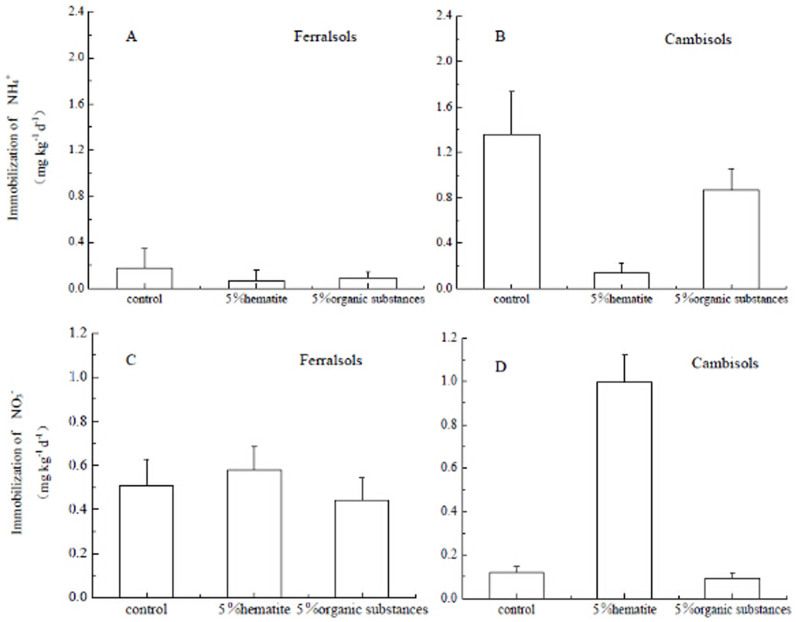
Effects of Fe oxide or organic substances addition on the immobilization of NO_3_^−^ and NH_4_^+^ (total of two immobilization rates including immobilization into recalcitrant and immobilization into labile organic N) (mg kg^−1^ d^−1^) in Ferralsols and Cambisols estimated by the ^15^N tracing model. Error bars represent standard deviation, n = 3.

NO_3_^−^ immobilization rates for Cambisols were not significantly affected by organic substances addition (Figure 3D) but they significantly increased by Fe oxide addition. NO_3_^−^ immobilization was also observed for the Ferralsol treatments, including the control and both Fe oxide and organic substances treatments. However, Fe and organic substances amendments did not significantly change NO_3_^−^ immobilization rates for Ferralsols compared to the unamended control (Figure 3C).

Gross NH_4_^+^ immobilization was four times higher for Cambisols than Ferralsols (Figure 3A, B). Hematite addition significantly decreased the NH_4_^+^ immobilization rate for Cambisols, and the organic substances treatment also showed a decreasing trend but the difference was not significant (p > 0.05). NH_4_^+^ immobilization rates for Ferralsols did not change in response to Fe oxide and organic substances addition.

**Figure 4:**
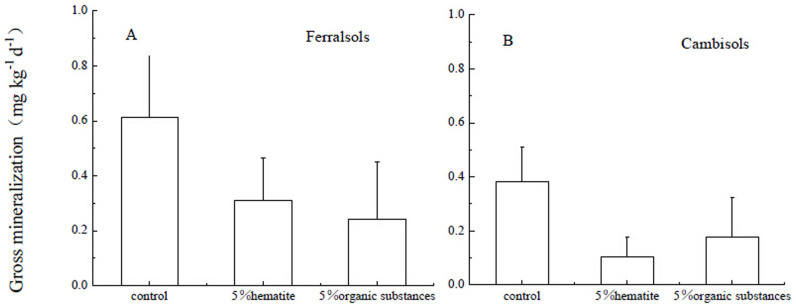
Effects of Fe oxide or organic substances addition on gross mineralization (mineralization of recalcitrant organic N and labile organic N to NH_4_^+^) (mg kg^−1^ d^−1^) in Ferralsols and Cambisols estimated by the ^15^N tracing model. Error bars represent standard deviations, n = 3.

Gross N mineralization rates did not differ significantly between Ferralsols and Cambisols in the Fe oxide and organic substance treatment (p > 0.05), although Fe oxide addition tended to decrease gross N mineralization for both soils (Figure 4).

### Ammonia-oxidizing bacterial (AOB) and ammonia-oxidizing archaeal (AOA) *amoA* gene copies

**Figure 5:**
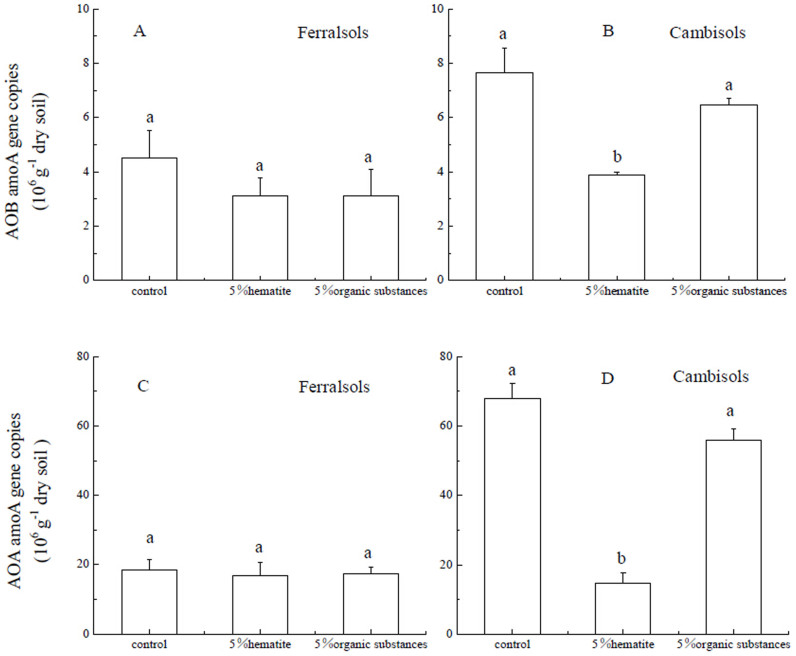
Effects of Fe oxide or organic substances addition on abundance of ammonia-oxidizing bacterial (AOB) and ammonia-oxidizing archaeal (AOA) *amoA* gene copies. Lowercase letters indicate statistically significant differences (p < 0.05). Error bars represent standard deviations, n = 3.

Abundance of AOB and AOA was estimated by quantifying their respective *amoA* gene copy numbers after pre-incubation (Figure 5). The organic substances treatment tended to decrease AOB abundance for both Ferralsols and Cambisols but results were not significant (p > 0.05). However, Fe oxide addition significantly decreased AOB *amoA* gene copy numbers by about 50% for Cambisols. For Ferralsols, Fe oxide addition also tended to decrease AOB abundance, but not significantly. The abundance of AOB was significantly higher for Cambisols than Ferralsols, but the difference was not significant after 5% Fe oxide addition.

AOA *amoA* gene copy numbers were significantly higher for Cambisols than Ferralsols (Figure 5). Fe oxide addition decreased AOA *amoA* gene copy numbers for Cambisols to a similar level in Ferralsols. For Ferralsols, Fe oxide addition also did not change AOA abundance significantly. Similar to AOB, AOA abundance showed a non-significant decreasing trend after the organic substances addition for both Ferralsols and Cambisols (p > 0.05).

## Discussion

While net nitrification was low for the highly acidic Ferralsols, gross autotrophic nitrification rates were about three times higher than net nitrification rates. The net nitrification rate was 8 times higher for weakly acidic Cambisols than highly acidic Ferralsols. However, the net nitrification rate decreased to a negative value for the weakly acidic Cambisols after Fe oxide addition. Results clearly showed that soil net nitrification rate was significantly inhibited by Fe oxide addition. However, only the evaluation of the associated individual gross N rates sheds light whether the oxidation to NO_3_^−^ or immobilization of NO_3_^−^ was responsible for this result.

Nitrification could be retarded by high amounts of Fe from pyrite (FeS_2_). Blaise^17^ reported that nitrification was inhibited by 40.3% with the addition of 10000 mg pyrite kg^−1^ soil at the end of a 30-day incubation. However, they were unable to identify the exact mechanism by which pyrite inhibits nitrification/immobilization. Furthermore, postulated that the effect of pyrite could be due to the toxic action of any one or a combination of the following: (1) sulphides^20^; (2) the oxidized forms of the sulphides, or (3) presence of Fe^2+^ ions^21^. Compared to their results, a stronger inhibition of net nitrification by 50000 mg hematite kg^−1^ soil was observed in the present study. However, the mechanisms they postulated for pyrite inhibition of nitrification did not seem to be applicable for the present study since S or sulphides were not involved. Therefore, other mechanisms must be responsible for ferric iron inhibition of nitrification.

The toxicity of Fe to nitrifying microorganisms may partly contribute to the depression of nitrification. A pioneer study by Meiklejohn^22^ indicated that small amounts of Fe (0.1–0.6 mg L^−1^) stimulated growth of nitrifying bacteria and increased the oxidation of NH_3_ to NO_2_^−^, whereas high concentrations of Fe (>112 mg L^−1^) was toxic to nitrifying bacteria. The present results clearly demonstrated that both *amoA* genes for the ammonia monooxygenase (AMO) of AOB and AOA decreased two or three times after Fe oxide addition to Cambisols, and the soil gross ammonia nitrification rate also decreased. Higher *amoA* gene copy numbers of AOB and AOA were observed for Cambisols than for Ferralsols. Since Fe oxide content of Ferralsols (50.1 mg kg^−1^ soil) was significantly higher than for Cambisols (16.6 mg kg^−1^ soil), AOB and AOA were likely inhibited by high Fe oxide content. However, Fe toxicity to AOB and AOA cannot explain the decrease of gross ammonia nitrification completely, because the average activity was estimated around 40 fmol NH_3_ cell^−1^ day^−1^ for AOA and AOB^23^,^24^. The gene copy numbers of AOB and AOA after Fe oxide addition still have potential nitrification up to 20 mg N kg^−1^ soil day^−1^.

The gross NO_3_^−^-N immobilization rate for Cambisols was 8 times higher in the 5% Fe oxide compared to the control treatment. In general immobilization of NO_3_^−^ is assumed to be assimilated into microbial biomass. However, higher NO_3_^−^-N immobilization induced by Fe oxide in the present soils can not be explained by the assimilation of NO_3_^−^-N into microbial biomass. This was supported by the fact that NH_4_, rather than NO_3_, is the preferred form of N for assimilation by soil microorganisms^25^,^26^,^27^. Further evidence was that the gene abundance of nitrifying microorganisms decreased after Fe oxide addition (Figure 5). Results from upland tropical forest soils in America also showed that microbial biomass ^15^N did not increase after NO_3_^−^ addition^28^. Positive charges on the soil surface may also increase NO_3_^−^-N immobilization under field conditions^29^,^30^,^31^. Anion exchange capacity (AEC) increased significantly after Fe oxide addition (Table 1). However, it cannot explain why NO_3_^−^-N concentrations kept decreasing with time after Fe oxide addition because most of the NO_3_^−^-N absorbed together with cations should have been released through ion exchange by the extractant (2 M KCl).

A potential mechanism of abiotic immobilization of NO_3_^−^ has been postulated, and the authors suggested that Fe plays a key role in NO_3_^−^-N immobilization by promoting organic N formation from inorganic N^16^. Shindo and Huang found that Mn oxide significantly promoted the abiotic formation of nitrogenous polymers in the pH range 4 to 8 within 24 hours^18^. Since Fe and Mn oxides have similar chemical properties, it is possible that Fe oxide also functions similarly and can thus promote organic N formation. The mechanism for this organic N formation may explain the N dynamics for the two soils evaluated in our study. In a recent study, Zhang et al. reported that the gross autotrophic nitrification ranged from 0 to 0.5 μg N g^−1^d^−1^ and the NO_3_^−^-N immobilization rate into organic N ranged from 0.11 to 1.71 μg N g^−1^d^−1^ in a subtropical acid forest soil^3^. Their results indicated high rates of NO_3_^−^-N immobilization into organic N were responsible for the N enrichment in humid subtropical forest soils which contained large amounts of Fe and Mn oxides. A higher net nitrification rate occurred for weakly acidic Cambisols than for highly acidic Ferralsols in our study, and the NO_3_^−^-N immobilization was higher for Ferralsols than for Cambisols. The Fe oxide content of Ferralsols (50.1 g kg^−1^ soil) was higher than for Cambisols (16.6 g kg^−1^ soil), which implied that the absence of nitrification in some highly acid soils may due to high amounts of NO_3_^−^-N immobilization stimulated by high Fe oxide concentrations.

Soil net nitrification, gross NH_4_^+^ oxidation, total gross NH_4_^+^ mineralization and NO_3_^−^-N immobilization did not change significantly by organic substances addition for both soils. The insignificant response of N transformations by C/N ratio or organic substances addition may be due to the short incubation period which was not enough to highlight possible effects.

Cambisols and Ferralsols formed from the same parent material in the same climate, but differing in pH. While net nitrification occurred for the slightly acidic Cambisols, no obvious net nitrification was observed for highly acidic Ferralsols. The soil net nitrification rate strongly decreased to a negative value after Fe oxide addition to the slightly acidic Cambisols. However, significant autotrophic nitrification was observed using ^15^N tracers for both soils. Fe oxide greatly increased NO_3_^−^-N immobilization, probably by promoting the abiotic formation of nitrogenous polymers. This suggests that the absence of net nitrification in some highly acid soils may due to high amounts of NO_3_^−^-N immobilization caused by the high Fe oxide content rather than the low pH.

## Methods

### Site description and soil sampling

The yellow soil samples (Ferralsols, Xanthic) were collected from a pine forest soil derived from a sandstone parent material in Jingyun Mountain, Chongqing, China (29°83′N,106°39′E). In this region, the annual mean temperature is 19.6°C and annual rainfall is 1611 mm. Middle subtropical evergreen broad-leaved forests characterize the vegetation of this area. Purple soils (Cambisols, Chromic) were collected at a nearby hill. The Ferralsols samples were highly weathered and experienced a long developing process. To the contrary, Cambisols were slightly weathered and have not experienced a long developing process because of soil erosion. Selected soil properties for two soils and each treatment were listed in Table 1.

Four field replicates were taken at each site to a 0–20 cm depth in October, 2012, with each replicate being composed of seven individual soil cores (diameter was 5.5 cm), which were pooled and homogenized to reduce heterogeneity. Samples for each of the four replicates per site were were air-dried and separated into two parts. One was ground to pass a 2-mm sieve and used to make subsamples for incubation, another was ground to pass a 1-mm sieve and used for chemical analyses.

### Preparation of Fe oxide and organic substances treatments

The precipitates of hematite were prepared from ferric chlorite of at least 98% purity, and solutions containing 100 g of FeCl_3_·6H_2_O/L were prepared. Ferric oxides were precipitated by the addition of NH_3_ to the FeCl_3_ solution until pH 7.0. The resultant suspension was thoroughly dialyzed for 24 hours at which time no further Cl^−^ and Fe^3+^ was detected in the external solution, followed by aging for 48 hours in a 160°C oven^32^. Obtained hematite was checked by X-ray powder diffractometer (XRD) (see Supplementary figure 1). The organic substances were collected from the organic layer of the Ferralsols, and sieved to pass a sieve <2 mm to remove coarse roots, mixed thoroughly and then air dried (20°C) for 3 days before use.

Subsamples were obtained by adding 5% hematite or 5% organic substances into Ferralsols or Cambisols, respectively. Six subsamples were obtained as follows: Ferralsols + 5% hematite, Ferralsols + 5% humic substances, Cambisols + 5% hematite and Cambisols + 5% humic substances. Subsamples were air-dried, and separated into two parts. One was ground to pass a 2-mm sieve and stored at 4°C for 2 months before use, another was ground to pass a 1-mm sieve and used for chemical analyses.

### Physical and chemical analyses

Soil pH was measured in a soil to water ratio of 1:2.5 (v/v) by a DMP-2 mV/pH detector (Quark Ltd, Nanjing, China). Total N (TN) and soil organic matter (SOM) contents were determined by a Macro Elemental Analyzer (Elementar Analysensysteme GmbH, Hanau, Germany). Total soil Fe was extracted with HNO_3_–HF–HClO_4_, and free Fe oxides were extracted with Na_2_S_2_O_4_–Na_3_C_6_H_5_O_7_–NaHCO_3_. Fe content was measured by atomic absorption spectrophotometry with a graphite furnace (GFAAS) using a model Z-8200 spectrophotometer. Soil cation exchange capacity (CEC) and anion exchange capacity (AEC) were determined after extraction with 0.0215 mol (NH_4_NO_3_) L^−1^ solution using the method described by Gillman & Sumpter^33^.

### Experimental design and ^15^N addition

The soil subsamples were adjusted to 60% water holding capacity (WHC) and pre-incubated for 7 days at 25°C. We employed a combination of ^15^N tracing experiments and full process-based N cycle models to quantify process-specific and pool-specific N transformation rates, which is the standard method for the quantification of N dynamics in soils^19^,^34^,^35^,^36^,^37^. For all samples, ^15^N tracing studies were carried out under controlled conditions. For the ^15^N tracing experiments, we employed two NH_4_NO_3_ treatments (each with three replications). In the first, NH_4_ (^15^NH_4_NO_3_) was labeled with ^15^N at 20 atom% excess, and in the second, NO_3_ (NH_4_^15^NO_3_) was labeled. For each soil sample, a series of 250-ml conical flasks was prepared, each containing 30 g of fresh soil. 2 ml of ^15^NH_4_NO_3_ or NH_4_^15^NO_3_ solution was added to each conical flask at a rate of 7.14 μmol N g^−1^ soil (50 μg NH_4_^+^-N g^−1^ soil and 50 μg NO_3_^−^-N g^−1^ soil). The soil was adjusted to 60% water-holding capacity (WHC) and incubated for 7 days at 25°C. All bottles were covered with polyethylene film punctured with needle holes to maintain aerobic conditions. The soils (three replications for each treatment) were extracted at 0.5, 24, 72, 120 and 168 hours after fertilizer application to determine the concentrations and isotopic composition of NH_4_^+^ and NO_3_^−^.

For isotopic analysis, NH_4_^+^ and NO_3_^−^ were separated by distillation with Mg oxide and Devarda's alloy^38^,^39^ (see Supplementary information). The isotopic composition of NH_4_^+^ and NO_3_^−^ were measured using an automated C/N analyzer coupled to an isotope ratio mass spectrometer (Europa Scientific Integra, UK). Simultaneous gross N transformations in soil were quantified using a process-based ^15^N tracing model^19^(see Supplementary information).

### DNA extraction & quantitative PCR assay

Right after pre-incubation, 4 replicates of each treatment were randomly selected to extract DNA and *amoA* genes were analyzed by quantitative PCR (qPCR). The DNA was extracted for three sub-samples from 0.50 g of soil with the FastDNA Spin Kit for soil (MP Biomedicals, United States), according to the protocol of the manufacturer. The quality and quantity of the DNA extracts were determined with a spectrophotometer (Nanodrop, PeqLab, Germany), and were pooled and stored at −20°C until use. Quantitative PCR of *amoA* genes was performed to estimate the abundance of the ammonia-oxidizing bacterial and archaeal communities, respectively (see Supplementary information).

### Statistical analyses

Data (measured or calculated) were subjected to one-way ANOVA and mean values were separated using Duncan's New Multiple Range Test at p < 0.05. All statistical analyses were performed by SPSS statistical package.

## Author Contributions

X.J. wrote the manuscript and carried out data analysis; X.X. and J.Z. prepared the experimental set-up and scientific protocols. X.X. and S.L. prepared the figures. C.M. provides the model for calculation and help in discussion. T.Z. did the calculation and data analysis. Z.C. help in discussion and data analysis. A.W. helped with discussion and language checking. All authors reviewed the manuscript.

## Supplementary Material

Supplementary Information supplementary information

## Figures and Tables

**Table 1:** Selected soil properties

Treatments	Texture	Parent material	pH	SOM	TN	CEC	AEC	Total Fe	Free Fe oxide	C/N
				(g/kg)	(g/kg)	(cmol/kg)	(cmol/kg)	(g/kg)	(g/kg)	
Ferralsols	Sandy loam	Purple sandstone	4.2	16.0 ± 0.34^b^	1.96 ± 0.01^a^	7.50 ± 0.86^b^	1.91 ± 0.23^b^	50.1 ± 4.33^a^	4.47 ± 0.47^b^	4.73
Ferralsols + 5% hematite			4.0	16.1 ± 0.69^b^	1.86 ± 0.06^a^	7.30 ± 0.77^b^	3.77 ± 0.17^a^		6.37 ± 0.84^a^	5.02
Ferralsols + 5% organic substances			4.1	20.9 ± 1.19^a^	1.85 ± 0.11^a^	7.37 ± 3.00^b^	2.24 ± 0.15^b^		4.68 ± 0.86^b^	6.55
Cambisols	Sandy loam	Purple sandstone	5.8	14.6 ± 1.62^b^	0.96 ± 0.10^b^	35.9 ± 2.20^a^	1.04 ± 0.47^c^	16.6 ± 1.21^b^	1.52 ± 0.10^c^	8.82
Cambisols + 5% hematite			5.4	14.3 ± 0.69^b^	0.81 ± 0.04^b^	33.3 ± 0.73^a^	1.93 ± 0.35^b^		5.84 ± 0.68^ab^	10.2
Cambisols + 5% organic substances			5.6	19.1 ± 1.39^a^	0.82 ± 0.09^b^	32.5 ± 3.29^a^	1.38 ± 0.22^c^		1.60 ± 0.23^c^	13.5

^a^SOM reprents soil organic matter; TN reprents total nitrogen; CEC reprents cation exchange capacity; AEC reprents anion exchange capacity.

^b^Values behind ± represent standard deviation.

^c^Mean values in a same column not followed by the same letter are different, p < 5%.

## References

[b1] Keller M., Kaplan W. A. & Wofsy S. C. Emissions of N_2_O, CH_4_ and CO_2_ from tropical forest soils. J. Geo. Res. 91, 11791–11802 (1986).

[b2] Dubinsky E. A., Silver W. L. & Firestone M. K. Tropical forest soil microbial communities couple iron and carbon biogeochemistry. Ecology 91, 2604–2612 (2010). 20957955 10.1890/09-1365.1

[b3] Yang W. H., Weber K. A. & Silver W. L. Nitrogen loss from soil through anaerobic ammonium oxidation coupled to iron reduction. Nature Geosci 5, 538–541 (2012).

[b4] Zhang J., Cai Z., Zhu T., Yang W. & Müller C. Mechanisms for the retention of inorganic N in acidic forest soils of southern China. Sci. Rep. 3, 2342 (2013). 23907561 10.1038/srep02342 PMC3731645

[b5] Tong D. & Xu R. Effects of urea and (NH_4_)_2_SO_4_ on nitrification and acidification of Ultisols from Southern China. J. Environ. Sci. 24, 682–689 (2012). 10.1016/s1001-0742(11)60832-2 22894103

[b6] Zhao W., Cai Z. & Xu Z. Does ammonium-based N addition influence nitrification and acidification in humid subtropical soils of China? Plant Soil 297, 213–221 (2007).

[b7] Zhao X. & Xing G. Variation in the relationship between nitrification and acidification of subtropical soils as affected by the addition of urea or ammonium sulfate. Soil Biol. Biochem. 41, 2584–2587 (2009).

[b8] Gosz J. R. & White C. S. Seasonal and annual variation in nitrogen mineralization and nitrification along an elevational gradient in New Mexico. Biogeochemistry 2, 281–297 (1986).

[b9] Vitousek P. M., Gosz J. R., Grier C. C., Melillo J. M. & Reiners W. A. A comparative analysis of potential nitrification and nitrate mobility in forest ecosystems. Ecol. Monogr. 52, 155–177 (1982).

[b10] Stark J. M. & Hart S. C. High rates of nitrification and nitrate turnover in undisturbed coniferous forests. Nature 385, 61–64 (1997).

[b11] Woodmansee R. & Duncan D. Nitrogen and phosphorus dynamics and budgets in annual grasslands. Ecology 61, 893–904 (1980).

[b12] Bengtsson G. & Bergwall C. Fate of ^15^N labelled nitrate and ammonium in a fertilized forest soil. Soil Biol. Biochem. 32, 545–557 (2000).

[b13] Priha O. & Smolander A. Nitrification, denitrification and microbial biomass N in soil from two N-fertilized and limed Norway spruce forests. Soil Biol. Biochem. 27, 305–310 (1995).

[b14] Nugroho R., Röling W., Laverman A., Zoomer H. & Verhoef H. Presence of Nitrosospira cluster 2 bacteria corresponds to N transformation rates in nine acid Scots pine forest soils. FEMS Microbiol. Ecol. 53, 473–481 (2005). 16329965 10.1016/j.femsec.2005.02.002

[b15] Bengtsson G., Bengtson P. & Månsson K. F. Gross nitrogen mineralization-, immobilization-, and nitrification rates as a function of soil C/N ratio and microbial activity. Soil Biol. Biochem. 35, 143–154 (2003).

[b16] Davidson E. A., Chorover J. & Dail D. B. A mechanism of abiotic immobilization of nitrate in forest ecosystems: the ferrous wheel hypothesis. Glob. Change Biol. 9, 228–236 (2003).

[b17] Blaise D., Amberger A. & Von Tucher S. Influence of iron pyrites and dicyandiamide on nitrification and ammonia volatilization from urea applied to loess brown earths (luvisols). Bio1. Fertil. Soils 24, 179–182 (1997).

[b18] Shindo H. & Huang P. Catalytic effects of manganese (IV), iron (III), aluminum and silicon oxides on the formation of phenolic polymers. Soil Sci. Soc. Am. J. 48, 927–934 (1984).

[b19] Mueller C., Rütting T., Kattge J., Laughlin R. & Stevens R. Estimation of parameters in complex ^15^N tracing models by Monte Carlo sampling. Soil Biol. Biochem. 39, 715–726 (2007).

[b20] Prasad R. & Reddy R. Effects of sulpha drugs on nitrification of urea in soil. Plant Soil 48, 11–16 (1977).

[b21] Goos R. & Ahrens W. Ammonium thiosulfate effect on herbicide longevity in soil. Agron. J. 84, 459–463 (1992).

[b22] Peretyazhko T. & Sposito G. Iron(III) reduction and phosphorous solubilization in humid tropical forest soils. Geochim. Cosmochim. Ac. 69, 3643–3652 (2005).

[b23] Hatzenpichler R. Diversity, physiology, and niche differentiation of ammonia-oxidizing archaea. Appl. Environ. Microb. 78, 7501–7510 (2012). 10.1128/AEM.01960-12 PMC3485721 22923400

[b24] Jiang X. *et al.* pH regulates key players of nitrification in paddy soils. Soil Biol. Biochem. 81, 9–16, 10.1016/j.soilbio.2014.10.025 (2015).

[b25] Jackson L. E., Schimel J. P. & Firestone M. K. Short-term partitioning of ammonium and nitrate between plants and microbes in an annual grassland. Soil Biol. Biochem. 21, 409–415 (1989).

[b26] Rice C. W. & Tiedje J. M. Regulation of nitrate assimilation by ammonium in soils and in isolated soil microorganisms. Soil Biol. Biochem. 21, 597–602 (1989).

[b27] Recous S., Mary B. & Faurie G. Microbial immobilization of ammonium and nitrate in cultivated soils. Soil Biol. Biochem. 22, 913–922 (1990).

[b28] Silver W. L., Herman D. J. & Firestone M. K. Dissimilatory nitrate reduction to ammonium in upland tropical forest soils. Ecology 82, 2410–2416 (2001).

[b29] Foster N., Morrison I., Yin X. & Arp P. Impact of soil water deficits in a mature sugar maple forest: stand biogeochemistry. Can. J. For. Res. 22, 1753–1760 (1992).

[b30] Houle D., Paquin R., Camiré C., Ouimet R. & Duchesne L. Response of the Lake Clair Watershed (Duchesnay, Quebec) to changes in precipitation chemistry (1988–1994). Can. J. For. Res. 27, 1813–1821 (1997).

[b31] Nemeth K. Recent advances in EUF research (1980–1983). Plant Soil 83, 1–19 (1985).

[b32] Harrison J. B. & Berkheiser V. E. Anion interactions with freshly prepared hydrous iron oxides. Clay. Clay Miner 30, 97–102 (1982).

[b33] Gillman G. P. & Sumpter E. A. Modification to the compulsive exchange method for measuring exchange characteristics of soils. Aust. J. Soil Res. 24, 61–66 (1986).

[b34] Huygens D. *et al.* Soil nitrogen conservation mechanisms in a pristine south Chilean Nothofagus forest ecosystem. Soil Biol. Biochem. 39, 2448–2458 (2007).

[b35] Mary B., Recous S. & Robin D. A model for calculating nitrogen fluxes in soil using ^15^N tracing. Soil Biol. Biochem. 30, 1963–1979 (1998).

[b36] Paterson E., Thornton B., Sim A. & Pratt S. Effects of defoliation and atmospheric CO_2_ depletion on nitrate acquisition, and exudation of organic compounds by roots of Festuca rubra. Plant Soil 250, 293–305 (2003).

[b37] Rütting T. *et al.* Functional role of DNRA and nitrite reduction in a pristine south Chilean Nothofagus forest. Biogeochemistry 90, 243–258 (2008).

[b38] Feast N. A. & Dennis P. F. A comparison of methods for nitrogen isotope analysis of groundwater. Chem. Geol. 129, 167–171 (1996).

[b39] Zhang J., Zhu T., Cai Z. & Müller C. Nitrogen cycling in forest soils across climate gradients in Eastern China. Plant Soil 342, 419–432 (2011).

