# Supplementary material for: Effects of Fe oxide on N transformations in subtropical acid soils
Source: Sci Rep. 2015 Feb 27;5:8615. doi: 10.1038/srep08615 (PMC4342567; doi:10.1038/srep08615)
Supplement: Supplementary Information [file srep08615-s1.pdf]

Title: Effects of Fe oxide on N transformations in subtropical acid soils

Xianjun Jiang[1*], Xiaoping Xin[1], Shiwei Li[1], Junchao Zhou[1], Tongbin Zhu[2], Christopher Müller[3,4], Zucong Cai[2], Alan L Wright[5]

[1]College of Resources and Environment, Southwest University, 2 Tiansheng Road, Beibei, Chongqing, 400715, China

[2]School of Geography Sciences, Nanjing Normal University, Nanjing 210047, China

[3]Department of Plant Ecology (IFZ), Justus-Liebig University Giessen, Heinrich-Buff-Ring 26, 35392 Giessen, Germany

[4]School of Biology and Environmental Science, University College Dublin, Ireland

[4]Everglades Research & Education Center, University of Florida, Belle Glade, FL 33430, United States

[*]E-mail: jiangxj@swu.edu.cn

Hematite was checked by X-ray powder diffractometer (XRD)

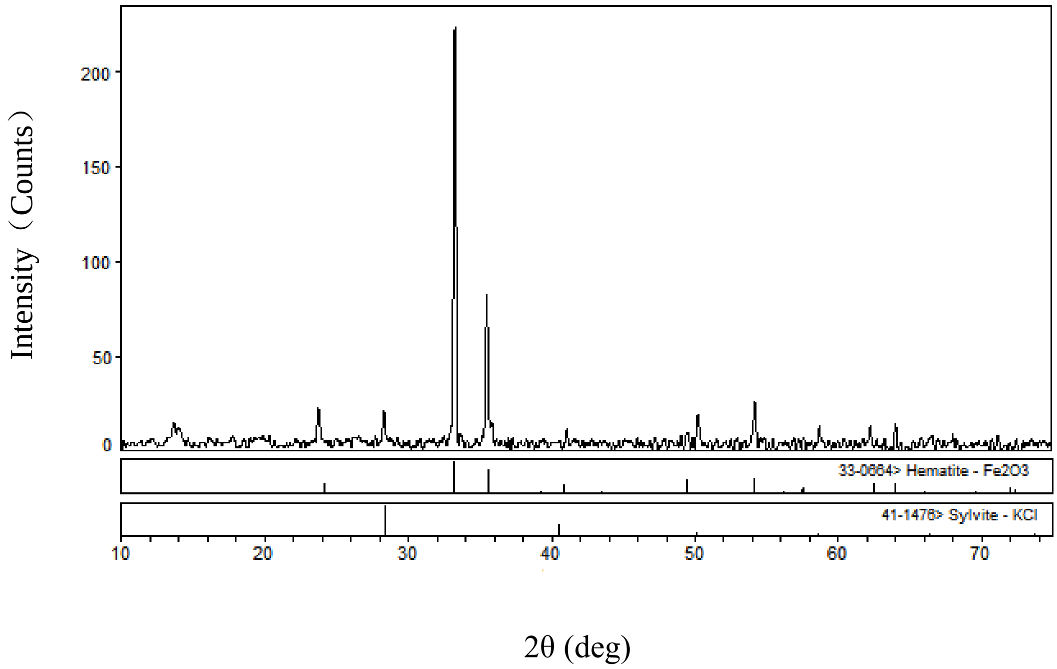

Supplementary figure 1, The XRD spectra for Hematite. The X-ray diffraction (XRD) patterns were collected by a Siemens D5000 powder diffractometer with Bragg–Brentano geometry equipped with a curved graphite monochromator in the diffracted beam arm and using Cu Kα radiation (λ= 0.15406). The XRD patterns of the samples were recorded in the range 2θ= 10°–90 °, using a step size of 0.02 ° and a counting time of 5 s/step.

^{15}N tracing model

The simultaneously-occurring gross N transformations in soil were quantified with a process-based 15N tracing model. The model analyzed 10 simultaneous gross N transformations. The transformation rates were calculated by zero-, first-order or Michaelis-Menten kinetics. Data supplied to the model included the concentrations and ^{15}N excess values (averages ± standard deviations) of NH_4^+ and NO_3^- from the two ^{15}N treatments. The model calculated gross N transformation rates by simultaneously optimizing the kinetic parameters for the various N transformations by minimizing the misfit between modeled and observed NH_4^+ and NO_3^- concentrations and their respective ^{15}N enrichments. To identify the most appropriate model that best described the measured N dynamics, we used the procedure described by Rütting et al.[1] ; the numbers of possible N transformations, kinetic settings and possible N pools were varied to find the best model. The final model was selected according to Akaike's Information Criterion (AIC), which takes into account the fit between observed and modeled data and the number of parameters[2]. Parameter optimization was carried out using the Metropolis algorithm (MCMC-MA; for further details on the algorithm see[3]. The misfit function between the observed and modeled data, *f*(**m**)(see eqt. 3 in reference [3]), takes into account the variance of the individual observations. Analyses using this parameter optimization concept in previous studies have shown that the mineralization of two conceptual organic-N pools produced realistic NH_4^+ dynamics[4]. The MCMC-MA routine was programmed in MatLab (Version 7.2, MathWorks Inc.), which calls models that are separately set up in

Simulink (Version 6.4, MathWorks Inc.). Initial concentrations of the mineral N pools (^{14}N and ^{15}N pool sizes) were determined according to Müller et al.[5]. Concentrations of NH_4^+ and NO_3^- were estimated for time zero by back-extrapolation of data at t = 0.5 h and t = 24 h. The difference between the applied NH_4^+ and the measured NH_4^+ was considered to be NH_4^+ that had been adsorbed shortly after N application to the NH_4^+ exchange sites (NH_{4ads}). The optimization procedure resulted in a probability density function (PDF) for each parameter, from which the parameter averages and standard deviations were calculated[3]. Each analysis was carried out with three parallel sequences to identify adequate iteration numbers. Based on the kinetic settings and the final parameters, the average N transformation rates were calculated over the entire period and expressed in units of mg N kg^{-1} soil day^{-1}.

Based on available studies of soil N cycling, this study focused on the total gross rates of mineralization (M_{Nrec}+M_{Nlab}), total nitrification (O_{NH4}+O_{Nrec}), autotrophic nitrification (O_{NH4}), NO_3^- (I_{NO3}) and NH_4^+ (I_{NH4_Nlab} + I_{NH4_Nrec}) immobilization, and DNRA (D_{NO3}). NH_4^+ = ammonium; NO_3^- = nitrate; NH_{4ads} = adsorbed NH_4^+; M_{Nrec}, mineralization of recalcitrant organic-N to NH_4^+; M_{Nlab}, mineralization of labile organic-N to NH_4^+; O_{NH4}, oxidation of NH_4^+ to NO_3^- (autotrophic nitrification); O_{Nrec}, oxidation of recalcitrant organic-N to NO_3^- (heterotrophic nitrification); I_{NO3}, immobilization of NO_3^- to recalcitrant organic-N; I_{NH4_Nlab}, immobilization of NH_4^+ to labile organic-N; I_{NH4_Nrec}, immobilization of NH_4^+ to recalcitrant organic-N; and D_{NO3}, dissimilatory NO_3^- reduction to NH_4^+.

DNA extraction & quantitative PCR assay

Right after pre-incubation, 4 replicates of each treatment were randomly selected to extract DNA and *amoA* genes were analyzed by quantitative PCR (qPCR). The DNA was extracted for three sub-samples from 0.50 g of soil with the Fast DNA Spin Kit for soil (MP Biomedicals, United States), according to the protocol of the manufacturer. The quality and quantity of the DNA extracts were determined with a spectrophotometer (Nanodrop, PeqLab, Germany), and were pooled and stored at -20 ℃ until use. Quantitative PCR of *amoA* genes was performed to estimate the abundance of the ammonia-oxidizing bacterial and archaeal communities, respectively. The primers *amoA*-1 F (5'-GGGGTTTCTACTGGTGGT-3') and *amoA*-2R (5'-CCCCTCKGSAAAGCCTTCTTC-3') were used for ammonia-oxidizing bacteria generating a 491 bp fragment; Arch-*amoA* F (5'-STAATGGTCTGGCTTAGACG -3') and Arch *amoA* R (5'-GCGGCCATCCATCTGTATGT -3') were used for ammonia- oxidizing archaea generating a 635 bp fragment[6]. Quantification was based on the fluorescence intensity of the SYBR Green dye and reactions for each sample were carried out in a Bio-Rad CFX-96 thermal cycler. The quantification of *amoA* genes was performed in a total volume of 25 μl reaction mixtures by using 12.5 μl of SYBR Premix Ex Taq™ as described by the suppliers (Takara Bio, Otsu, Shiga, Japan), 0.25 μl of each primer (50 μm), 1 μl of soil DNA template, with a final content of 1–10 ng in each reaction mixture, and 11 μl ddH_2O.The fragments for the AOB and AOA were both amplified using an initial denaturation step at 95 ℃ for 3 min, followed by 35 cycles of 30s at

95 ℃, 30s at 55 ℃, 30s at 72 ℃ for AOB, and 45 s at 72 ℃ for AOA for the collection of fluorescence data. All reactions were finished with a melting curve starting at 65 ℃ with an increase of 0.5 ℃ up to 95 ℃ to verify amplicon specificity. The PCR reaction runs had an efficiency of 90 % and 94 % for the AOB and AOA, respectively. Standard curves for the AOB and AOA were obtained using serial dilutions of linearized plasmids (pGEM-T, Promega) containing cloned *amoA* genes amplified from environmental clones ($r^2 > 0.995$ for both standard curves).

References

1. Rütting, T. et al. Functional role of DNRA and nitrite reduction in a pristine south Chilean Nothofagus forest. *Biogeochemistry* **90**, 243-258 (2008).
2. Cox, G.M. *et al.* Towards the systematic simplification of mechanistic models. *Ecol. Model*. **198**, 240-246. (2006)
3. Müller, C., Rütting, T., Kattge, J., Laughlin, R. & Stevens, R. Estimation of parameters in complex 15N tracing models by Monte Carlo sampling. *Soil Biol. Biochem*. **39**, 715-726 (2007).
4. Huygens, D. et al. Soil nitrogen conservation mechanisms in a pristine south Chilean< i> Nothofagus</i> forest ecosystem. *Soil Biol. Biochem*. **39**, 2448-2458 (2007).
5. Müller, C., Stevens, R. & Laughlin, R. A ^{15}N tracing model to analyse N transformations in old grassland soil. *Soil Biol. Biochem.* **36**, 619-632 (2004).
6. Francis, C. A., Roberts, K. J., Beman, J. M., Santoro, A. E. & Oakley, B. B.

Ubiquity and diversity of ammonia-oxidizing archaea in water columns and sediments of the ocean. *Proc. Natl. Acad. Sci.***102**, 14683-14688 (2005).
